# Necrotizing Fasciitis Resulting from an Anastomotic Leak after Colorectal Resection

**DOI:** 10.1155/2018/8470471

**Published:** 2018-09-16

**Authors:** Anthony Nagib, Chauniqua Kiffin, Eddy H. Carrillo, Andrew A. Rosenthal, Rachele J. Solomon, Dafney L. Davare

**Affiliations:** ^1^Nova Southeastern University, College of Osteopathic Medicine, 3301 College Avenue, Fort Lauderdale, FL 33314, USA; ^2^Memorial Regional Hospital, Division of Acute Care Surgery and Trauma, 3501 Johnson Street, Hollywood, FL 33021, USA; ^3^Memorial Regional Hospital, Office of Human Research, 4411 Sheridan Street, Hollywood, FL 33021, USA

## Abstract

One of the most feared complications in colorectal surgery is an anastomotic leak (AL) following a colorectal resection. While various recommendations have been proposed to prevent this potentially fatal complication, anastomotic leaks still occur. We present a case of an AL resulting in a complicated and fatal outcome. This case demonstrates the importance of high clinical suspicion, early recognition, and immediate management.

## 1. Introduction

Colorectal anastomotic leaks (AL) are a common, yet serious complication of colorectal resections, with occurrence rates of 2–21% and mortality rates of 3–33% [1–6]. AL may have various clinical presentations throughout a patient's postoperative course. Due to its nonspecific presentation, few clinical criteria exist to define the development of an AL [2, 3, 7, 8]. Thus, a high index of suspicion and clinical judgment are paramount to the early recognition and prevention of fatal outcomes. We present a case of an AL with a unique presentation that occurred 8 years after a colorectal resection.

## 2. Case Presentation

**Figure 1:**
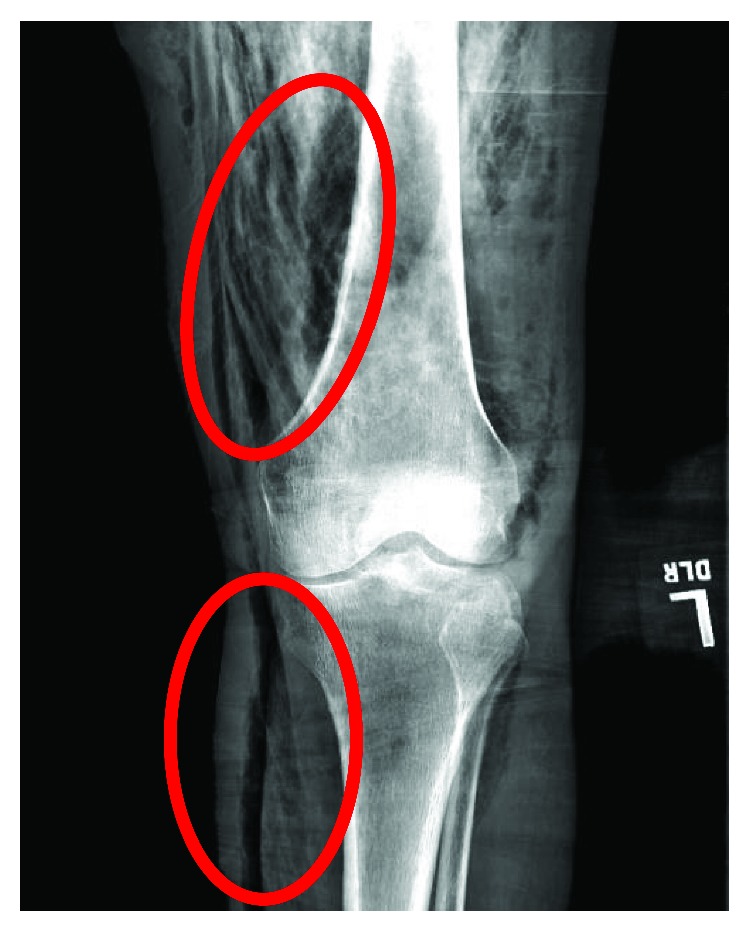
AP radiograph of the left lower extremity demonstrating subcutaneous emphysema (red ovals).

**Figure 2:**
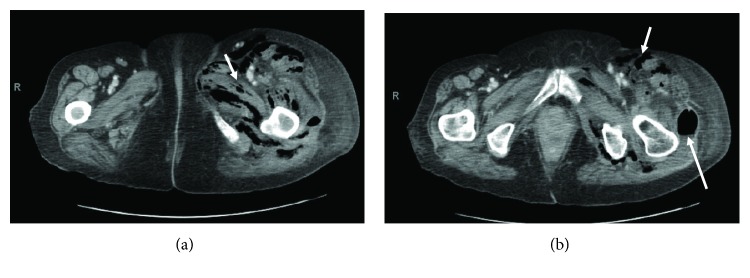
Axial CT images with IV contrast of the lower pelvis (a and b) demonstrating extensive subcutaneous emphysema consistent with necrotizing fasciitis around the left femur (short arrows). Note the air filled abscess cavity (b) filled along the posterior aspect of the left hip (long arrow).

**Figure 3:**
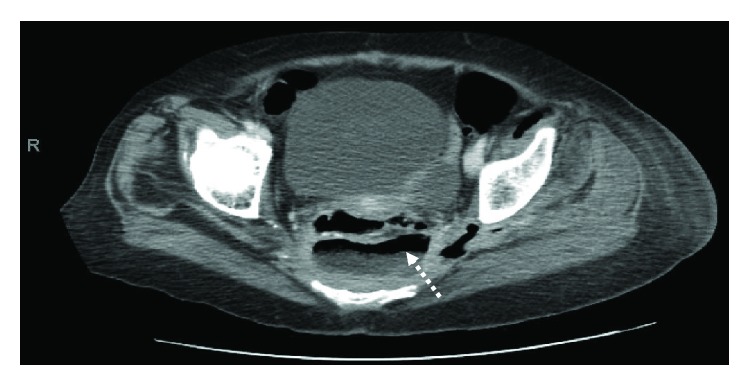
Axial CT images with IV contrast of the pelvis showing the extraperitoneal abscess (dotted arrow) derived from a previous colorectal anastomosis.

A 76-year-old female presented to the emergency department with complaints of the left thigh and hip pain and swelling for five days. She reported having a history of chronic left leg sciatic pain that contributed to a fall two days prior to the onset of these symptoms. Her past medical history was significant for colon cancer requiring a low anterior resection, which is eight years ago. The patient was noted to be confused and tachycardic. She was afebrile but had leukocytosis of 14,000. On physical examination, she was noted to have a significant crepitus to the left thigh and knee. Radiographs of the left leg confirmed subcutaneous emphysema consistent with necrotizing fasciitis (Figure 1). Prior to surgical consultation, the patient also received a pelvic computed tomography (CT) scan to evaluate for hip fractures. This further confirmed the necrotizing fasciitis (Figures 2(a) and 2(b)) but also identified a collection in the presacral space (Figure 3) that communicated to the left leg through the left sciatic notch, which is consistent with an AL. The patient was immediately taken to the operating room for debridement of the thigh and diverting colostomy.

An exploratory laparotomy with diverting colostomy was created to control ongoing contamination of the leg. Intra-abdominally, there were no abnormal findings, which is consistent with the extraperitoneal nature of the disease process. The decision, at this point, was to access the extraperitoneal collection through interventional radiology so as to minimize intra-abdominal contamination. After the colostomy was completed, the left thigh and hip were incised revealing a significant amount of feculent and purulent drainage. Necrotic, nonviable tissue was debrided down towards the knee, and the wound was left open and dressed. The patient was septic during the procedure and remained septic postoperatively. After an initial discussion with the patient's family, the plan was to perform percutaneous drainage of the presacral abscess postoperatively and obtain an orthopedic consultation as the hip joint was actively infected from the AL.

Recommendations by orthopedic and trauma consultants were that the patient would initially need an above the knee amputation due to the significant soft tissue loss and function from the extensive debridement. Furthermore, their concern was that this patient may ultimately need disarticulation of the left hip with potential hemipelvectomy if severe and recurrent osteomyelitis developed.

The patient's family ultimately decided to withdraw care, and the patient died in the hospital on day three.

## 3. Discussion

Colorectal anastomotic leaks have an incidence that varies from 2–30% [3, 4, 9]. The development of this complication leads to increased lengths of hospital stay, significant morbidity, and mortality rates of 6–32% [2, 4, 9]. There are several studies that have identified risk factors that contribute to the breakdown of a colorectal anastomosis. These include operative duration, male sex, diabetes, tobacco use, obesity, and immunosuppression [3, 9]. In addition, the type of anastomosis created can be a risk factor for its break down. For example, low anterior resections have been seen to have higher rates of anastomotic breakdown when compared to more proximal anastomoses [1, 6]. Some studies found that an anastomosis within 7 cm of the anal verge was an independent risk factor for AL [1, 8].

Presentation of ALs can vary in time of development and in symptomology. Anastomotic leaks can present as early as within the first postoperative week or as late as several years after the operation, as seen in our case. Early leaks, those presenting within 5 days of surgery, will present with nonspecific findings of pain, fever, tachycardia, and leukocytosis. It is imperative to suspect and identify this complication as early as possible. The utilization of CT scan or water-soluble contrast enema can assist in determining the presence of an anastomotic breakdown and can guide the surgeon in appropriate management [2, 7]. Leaks that occur after 5 days can also present with nonspecific findings, with a wide range of signs and symptoms. Examples include low-grade fever, prolonged ileus, urinary symptoms, and diet intolerance. Utilization of the aforementioned diagnostic studies can guide management.

Timing of ALs can affect the presentation as well as the location of the anastomotic breakdown. Extraperitoneal leaks are less likely to present with a severe septic picture, when compared to intraperitoneal leaks. An extraperitoneal leak could have an insidious onset and therefore be discovered after harm has already occurred, as seen in our case [2]. On the other hand, an intraperitoneal leak usually presents earlier with a clinical picture of peritonitis and sepsis due to peritoneal contamination.

In this case, the location of the anastomotic breakdown leads to extraperitoneal drainage into the sciatic canal with subsequent contamination of the left lower extremity and necrotizing fasciitis. This is a very rare occurrence with limited research and case studies discussing this type of presentation. On exam, the patient painted a clinical picture of necrotizing fasciitis, which was thought to be related to a recent trauma. However, her history of chronic left-sided sciatica may have been an indication of a very small persistent leak that over time contributed to her overall presentation.

The management of ALs should begin before surgery. If possible, preoperative optimization should be considered; this includes smoking cessation, weight loss, and improving nutritional status. Intraoperatively, the meticulous surgical technique must be utilized to ensure that the anastomosis is free of tension and remains well-vascularized. Consideration of a proximal stoma should be entertained in complex surgical cases to protect the anastomosis. Evaluation of the anastomosis can also include the use of air-leak testing, which is a common intraoperative practice. This involves manual obstruction proximal to the anastomosis while the peritoneal cavity is filled with saline. The introduction of the proctoscope and colorectal insufflation of air should create bubbling in the presence of an anastomotic breakdown. Multiple studies have shown that air-leak testing decreases the rate of leaks due to early detection. In one study, 77% of anastomoses that tested positive on air-leak testing had a confirmed leak postoperatively [7].

Postoperative management of an AL can either be nonsurgical or surgical. Nonoperative management is utilized when the leak is a localized abscess. These events can be treated with percutaneous drainage and antibiotics. In the presence of sepsis and peritoneal contamination, abdominal reexploration is warranted with the creation of a proximal diverting stoma. The choice of operative management is done on a case-by-case basis with clinical judgment being the ultimate determining factor. Of note, simple suture repairs of an AL are often unsuccessful and have been shown to cause further disruption of the anastomotic breakdown [2].

With the incidence of colorectal ALs as high as 30% in some studies, its recognition and management are of utmost importance [3, 4, 9]. Unfortunately, there is a paucity of literature providing clinicians with precise definitions and algorithms for recognizing and managing this potentially lethal complication. Computed tomography can be very helpful in both diagnosing and planning management of an AL. In this case, the utilization of CT imaging was very helpful in identifying the cause of this patient's presentation. However, under different circumstances, the patient may have undergone an emergent debridement without such imaging. The identification of stool drainage from the leg and the history of colorectal surgery should be a red flag for the anastomotic breakdown, prompting intervention.

## 4. Conclusion

This case highlights that ALs can occur at any time following colorectal surgery. In addition, this case demonstrates a unique presentation of an AL. In our patient, the presentation was 8 years after her original surgery. Furthermore, it is difficult to ascertain the cause for the delayed breakdown. The patient's age, nutritional status, and site of resection and anastomosis are potential contributing factors to this complication. It is important to consider an AL as a potential differential diagnosis in any patient with a history of colorectal surgery presenting with abdominal pain, fever, and leukocytosis.

ALs are a significant complication with severe consequences. In our case, it resulted in mortality due to delay in both presentation and diagnosis. Early identification and high clinical suspicion are critical to mitigating morbidity and mortality. Furthermore, the clinician must keep this potentially lethal complication in mind, even in the patient with a remote history of colorectal surgery. In all, the most reliable way of preventing morbidity and mortality from AL is by having a high index of suspicion to ensure early detection, workup, and intervention.
